# Porous acid–base hybrid polymers for enhanced NH_3_ uptake with assistance from cooperative hydrogen bonds†

**DOI:** 10.1039/d3ra05346f

**Published:** 2023-10-02

**Authors:** Xiaoyan Luo, Yibang Liu, Mingxing Li, Renhui Ling, Ling Ye, Xuegong Cao, Congmin Wang

**Affiliations:** a Xiamen Key Laboratory of Optoelectronic Materials and Advanced Manufacturing, Key Laboratory of Molecular Designing and Green Conversions (Fujian Province University), College of Materials Science and Engineering, Huaqiao University Xiamen 361021 P.R. China chemistrylxy@163.com; b Department of Chemistry, Center of Chemistry for Frontier Technologies, Zhejiang University Hangzhou 310027 P. R. China chewcm@zju.edu.cn

## Abstract

Carboxylic acid-modified materials are a common means of achieving efficient NH_3_ adsorption. In this study, we report that improved NH_3_ adsorption capacity and easier desorption can be achieved through the introduction of substances containing Lewis basic groups into carboxylic acid-modified materials. Easily synthesized mesoporous acid–base hybrid polymers were constructed with polymers rich in carboxylic acid and Lewis base moieties through cooperative hydrogen bonding interactions (CHBs). The hybrid polymer PAA–P4VP presented higher NH_3_ capacity (18.2 mmol g^−1^ at 298 K and 1 bar NH_3_ pressure) than PAA (6.0 mmol g^−1^) through the acid–base reaction and the assistance from CHBs with NH_3_, while the NH_3_ desorption from PAA–P4VP was easier for the reformation of CHBs. Based on the introduction of CHBs, a series of mesoporous acid–base hybrid polymers was synthesized with NH_3_ adsorption capacity of 15.8–19.3 mmol g^−1^ and high selectivity of NH_3_ over CO_2_ (*S*_NH_3_/CO_2__ = 25.4–56.3) and N_2_ (*S*_NH_3_/N_2__ = 254–1068), and the possible co-existing gases, such as SO_2_, had a lower effect on NH_3_ uptake by hybrid polymers. Overall, the hybrid polymers present efficient NH_3_ adsorption owing to the abundant acidic moieties and CHBs, while the concomitant Lewis bases promote NH_3_ desorption.

## Abstract 2

Porous acid–base hybrid polymers present improved properties of NH_3_ uptake and desorption compared with the pure acid polymer owing to the reversible cooperative hydrogen bonding interactions between carboxylate acid and Lewis base moieties.

## Graphical abstract

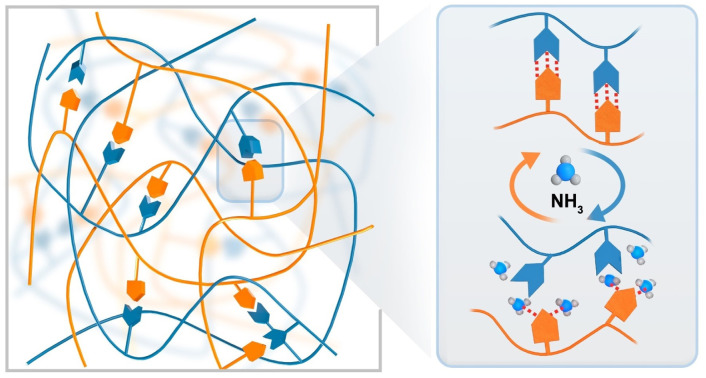

## Introduction

Ammonia (NH_3_) is indispensable in our lives today for producing artificial fertilizers and several military and commercial products, including explosives, refrigerants, pharmaceuticals, and synthetic fibers. NH_3_ is also a potential fuel, providing a way to store and transport hydrogen owing to its exploitable energy density. However, the unavoidable leakage of NH_3_ during its utilization has huge adverse impacts on the environment and human health. In these applications associated with the critical risks of this gas, effective adsorbents possessing the ability to store more NH_3_ have been attracting substantial attention.

Porous materials with high surface areas, such as active carbon^1^ and zeolites,^2–4^ have been used as NH_3_ adsorbents; however, they suffer from relatively low affinity and limited capacity for NH_3_. Simultaneously, porous materials, including metal–organic frameworks (MOFs),^5,6^ porous organic polymers (POPs),^7^ covalent organic frameworks (COFs),^8^ and hydrogen-bonded organic frameworks (HOFs),^9^ were developed as efficient NH_3_ adsorbents owing to their highly porous nature, strong stability, and designable abundant binding sites.^10^ The modification of porous materials with functionalized moieties was expected to result in superior adsorbed amounts. Functionalized UiO-66-A/B/C^11^ and UiO-66-ox synthesized *via* post-synthetic modification with free carboxylic acids indicated their positive utilization in NH_3_ capture,^12^ likewise the NH_3_ adsorption performance of Zr-based UiO-66 analogues.^13,14^ Various Brønsted acidic groups, such as –CO_2_H and –SO_3_H, were used to functionalize the water-stable framework UiO-66 and were reported with improved NH_3_ capacity.^15^ Acid-loaded porphyrin-based MOFs^16^ for capturing NH_3_ show remarkable stability and the isoreticular porphyrin-based MOFs^17^ were reported with rod-like secondary building units of Brønsted acid bridging hydroxyl groups for NH_3_ sorption. Similarly, the zirconium-based MOF, NU-300 with free Brønsted acid sites benefited the binding of NH_3_ even at low pressures.^18^ Recently, carboxylic-functionalized mesoporous copolymers PDVB-xAA were developed for fast, highly efficient, selective, and reversible NH_3_ adsorption.^19^ These studies revealed that NH_3_ capture relies on the interplay of the functional groups, especially the –COOH of adsorbents.

It was found that the COFs modified with weak acid groups and hydrogen bonding interactions presented more efficient NH_3_ uptake than strong acid-functionalized COFs. BBP-5, a COF with carboxylate acid group showed more efficient and reversible NH_3_ uptake than PPN-6-SO_3_H due to the existence of cooperative hydrogen bonds (CHBs) along with the acid-Lewis base interactions between carboxylic acid and NH_3_, which indicated that multiple chemical interactions were optimized to single strong interactions.^20^ Similarly, urea-functionalized Zn-MOFs with an increased number of hydrogen bonds^21^ were also reported to have outstanding NH_3_ adsorption. HOFs, mainly constructed by the self-assembly of organic molecules *via* intermolecular hydrogen bonding interactions, were explored as a potential corrosive gas trapping agent because of their abundant CHBs and porous structure. Jancik^22^ explored UNAM-1 constructed of hydrogen-bonded frameworks, which achieved the porosity required for reversible SO_2_ uptake. Kang^9^ reported a hydrogen-bonded network KUF-1 for NH_3_ capture with a sigmodal adsorption isotherm and achieved the NH_3_ capacity of 6.67 mmol g^−1^ at 1 bar. They found that the design of an adsorbent/absorbent with a flexible hydrogen bonding network presented superior NH_3_ capacity and desorption regeneration. However, the refined design and cost-intensive synthesis of MOFs, COFs, and HOFs are very real problems against their application.

It was found that the hydrogen bonding interactions between the ionic liquids (ILs) and NH_3_ were the key to the significant increase in NH_3_ capacity according to Palomar's work.^23^ Therefore, protic ILs^24,25^ and ILs substituted with hydroxyl groups,^23,26^ and poly ionic liquids (PILs) served as adsorbents of ammonia.^27,28^ In our previous work,^29,30^ amidine and pyridine-based protic ILs constructed with CHBs presented sigmodal isotherms, and the results indicated a high NH_3_ capacity of 7.5–9.3 mmol g^−1^ at 1 bar, 30 °C; furthermore, the threshold pressure could be regulated through the CHBs. Deep eutectic solvents (DESs), known as new ILs analogues, consist of hydrogen bond acceptors (HBA) and hydrogen bond donors (HBD) in suitable molar ratios based on hydrogen bonding interactions and other intermolecular noncovalent interactions.^31–38^ It has been proposed that DESs are very promising NH_3_ absorbents through Lewis acid–base and hydrogen bonding interactions.^39^ For instance, choline chloride-composed DESs were developed for NH_3_ absorbents,^40–42^ and choline chloride/resorcinol/glycerol (1 : 3 : 5) presented the NH_3_ absorption capacity of 13 wt% at 313.2 K and 0.1 MPa for the internal hydrogen-bonded network of DESs.^40^ Alcohol, phenol,^43,44^ and sugar^45^ were also selected as components of DESs by taking advantage of their hydroxyl groups for effective NH_3_ absorption. Protic DESs with NH_4_SCN, ethylamine hydrochloride and ethanolamine hydrochloride as HBAs were reported as excellent NH_3_ absorbents through strong hydrogen bonding interactions,^46,47^ and the influences of HBA of EDSs on the NH_3_ absorption performances were systematically investigated. According to the weak acidity of azole, azole-based DESs were also used as NH_3_ absorbents;^48–50^ it was found that the greater acidity of the azole benefited the NH_3_ capacity but went against the reversibility.^51^ Recently, it was reported that DESs involving metal chlorides including LiCl,^52^ M(II)Cl_2_,^53,54^ and M(III)Cl_3_,^53^ could achieve greater NH_3_ capacity for the coordination of NH_3_ with metal and hydrogen bonding interactions. For instance, it was found that the CHBs-rich ILs/DESs had a good trapping effect on NH_3_, while the NH_3_ absorption capacity would be enhanced by 18.1–36.9% when a small amount of metal chlorides was added to Res/EG (1 : 2) DES.^54^

**Fig. 1:**
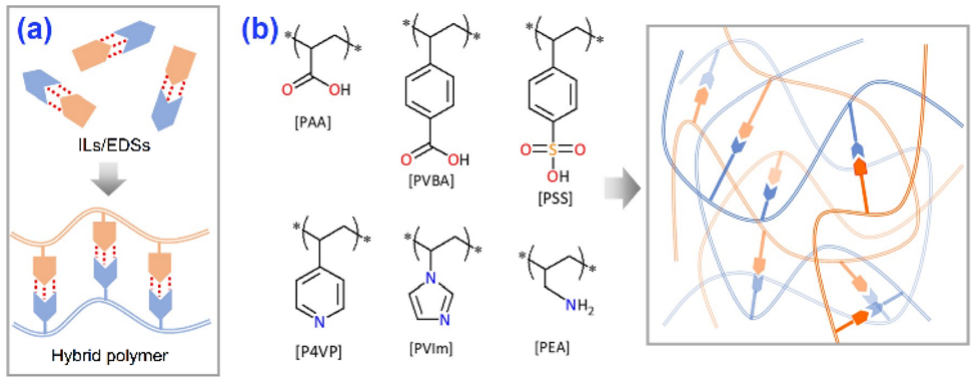
(a) A diagram showing the idea of developing acid–base hybrid polymers for NH_3_ uptake. (b) Structures of the agents used for synthesizing hybrid polymers.

The reports of sorbents substituted with carboxylic acid for efficient NH_3_ adsorption and the utilization of hydrogen bonds to improve NH_3_ desorption inspired us to construct self-assembled hybrid polymers based on the formation of CHBs between carboxylate acids and Lewis bases (Fig. 1a), which would enhance the affinity for NH_3_ as well as promote NH_3_ desorption by the reconstruction of CHBs. To obtain this target, various acid–base hybrid polymers (Fig. 1b) were explored to analyze the effects of the interactions between acidic and basic groups on NH_3_ uptake.

## Results and discussion

The reagents, preparation and characterization of hybrid polymers, and the NH_3_ adsorption and desorption experiments are described in the ESI File† in detail.

### The characterization of hybrid polymers

To explore the feasibility of the utilization of CHBs in enhancing the NH_3_ uptake by acid–base hybrid polymers, PAA and PVBA with carboxylic acid groups were hybridized with P4VP/PVIm containing Lewis base groups to synthesize hybrid polymers as NH_3_ adsorbents. The CHBs were further proposed from the optimized structures calculated through the DFT method as shown in Fig. S2.† The interaction energies between these acid and basic moieties were between −40.23 and −46.90 kJ mol^−1^ as listed in Table 1, which allows the possibility to self-assemble the acid–base hybrid polymers. For PAA–P4VP, the interactions between PAA and P4VP were calculated through propionic acid and 4-ethylpyridine, the reaction enthalpy was −40.23 kJ mol^−1^ and the Gibbs free energy was −1.28 kJ mol^−1^, which indicate the possibility of interaction between PAA and P4VP.

**Table 1:** The interaction energies between acidic and basic groups and the NH_3_ desorbed active energy from the TPD test

Entry	Complex	Interaction energy^a^ (kJ mol^−1^)	TDP peak (°C)	Desorbed active energy^b^ (kJ mol^−1^)
1	PAA–P4VP	−40.23	78	104.0
2	PAA–PVIm	−46.90	90	107.6
3	PVBA–P4VP	−40.05	75	103.2
4	PVBA–PVIm	−46.75	87	106.8
5	PAA	—	112	114.3

a The interaction energies between acidic and basic groups were obtained through DTF calculations on the B3LYP basis set at the 6-31G++ level.

b The active energies of NH_3_ desorption were calculated based on the Redhead method^55^ according to the desorption temperature from NH_3_-TPD.

The elemental contents of C, N, and H listed in Table S1† correspond with the theoretical data calculated from the molar ratio of acid and base moieties of 1 : 1, indicating that the hybrid polymers self-assemble in the equimolar reaction of –COOH and Lewis base. The IR spectra of acid–base hybrid polymers in Fig. S3† exhibit the changes in the vibration of C=O as compared with acid polymers for the formation of CHBs. The IR spectra of PAA and PAA–P4VP in Fig. S3a† show that the stretching vibration of –COOH at 1698 cm^−1^ was blue shifted to 1713 cm^−1^ with the hybridization with P4VP (marked with a star). According to the 2D correlation IR spectra in Fig. S4,† the absorption of *ν*(COOH) was correlated with the vibration of the pyridine group at about 1600 cm^−1^ (marked with a red star), which indicates the interaction between PAA and P4VP.

**Fig. 2:**
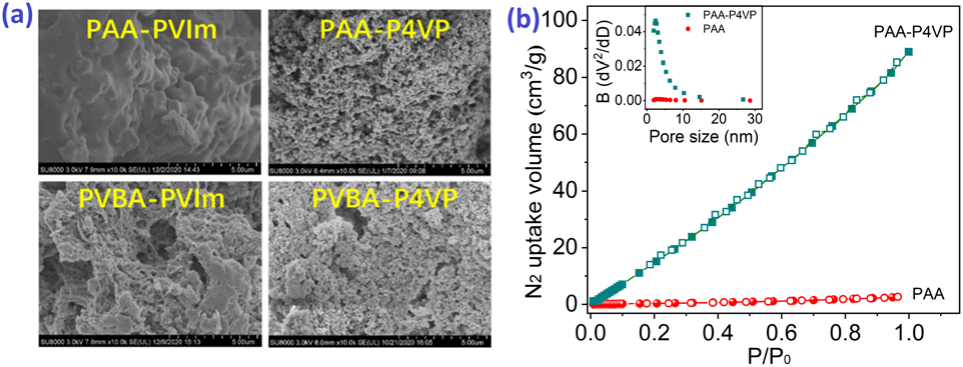
(a) The SEM images of hybrid polymers. (b) The N_2_ adsorption isotherm curve at 77 K and the pore distribution (inner picture) of PAA and PAA–P4VP.

The SEM images in Fig. 2a present the mesoporosity of these hybrid polymers. The BET surface areas of hybrid polymers according to N_2_ adsorption at 77 K in Fig. 2b and S5† range from 11.2 to 23.6 m^2^ g^−1^ (Table 2) and their pore size distribution is 2–30 nm, which explains the mesoporous structure of these hybrid polymers. On comparison of the SEM images and N_2_ adsorptions of nonporous PAA in Fig. S6b† and 2b, it was concluded that the porosity of PAA–P4VP was due to the multiple interactions between PAA and P4VP.

**Table 2:** The BET surface areas and gas uptake capacities of hybrid polymers and PAA

Entry	Complex	*S*_BET_ a (m^2^ g^−1^)	Gas capacity (mmol g^−1^)			Selectivity^d^	
			NH_3_^b^	N_2_^c^	CO_2_^c^	NH_3_/N_2_	NH_3_/CO_2_
1	PAA–P4VP	23.6	18.2	0.0445	0.396	408	54.0
2	PAA–PVIm	11.2	19.3	0.0181	0.398	1068	48.6
3	PVBA–P4VP	23.0	18.4	0.0723	0.557	254	33.0
4	PVBA–PVIm	23.6	15.8	0.0478	0.622	331	25.4
5	PAA	0.7	6.0	—	—	—	—

a The specific surface areas were calculated by the Brunauer–Emmett–Teller equation according to the N_2_ adsorption–desorption isotherm at 77 K.

b The NH_3_ uptake was operated under the self-made device at 25 °C.

c The N_2_ and CO_2_ adsorptions were detected *via* Micromeritics BAFLEX surface characterization measurements at 25 °C.

d The selectivity of NH_3_ over N_2_ (CO_2_) was calculated based on the capacity of NH_3_ divided by the capacity of N_2_ (CO_2_).

### NH_3_ uptake of PAA–P4VP *vs.* pure PAA

**Fig. 3:**
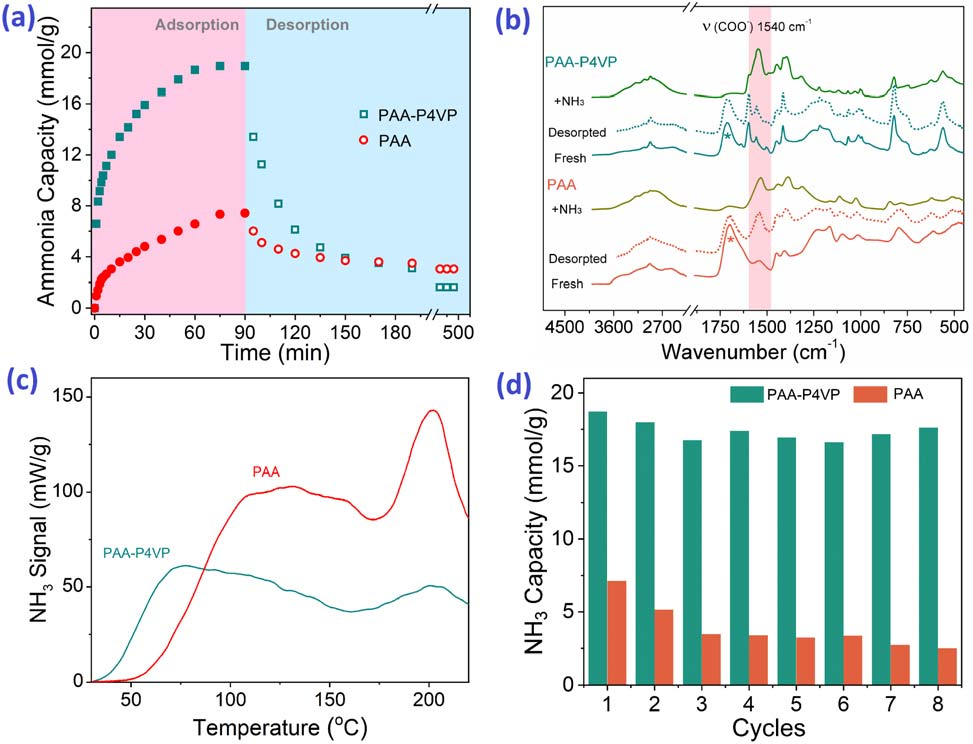
(a) The NH_3_ uptake and residual capacity of PAA and PAA–P4VP with time. (b) FT-IR spectra of fresh, NH_3_ saturated, and recovered PAA and PAA–P4VP. (c) NH_3_-TPD curves of PAA and PAA–P4VP with temperature increase ratio of 2 °C min^−1^ to 220 °C. (d) 8 consecutive NH_3_ adsorption–desorption results for PAA and PAA–P4VP. NH_3_ uptake at 25 °C, 1 bar. NH_3_ desorption at 80 °C under vacuum.

It was reported that materials rich in CHBs are suitable for efficient NH_3_ uptake; thus, hybrid polymers were used for NH_3_ uptake. Interestingly, PAA–P4VP presents a higher NH_3_ uptake capacity of 18.2 mmol g^−1^ than PAA (6.0 mmol g^−1^) from Fig. 3a, and the NH_3_ trapped in PAA–P4VP would be released feasibly. From the reported data according to the properties of DESs, ILs, and acid-modified MOFs listed in Table S2,† the hybrid polymer showed excellent NH_3_ uptake capacity and mild desorption conditions. The comparison of the IR spectra of fresh and NH_3_ saturated samples in Fig. 3b indicates that –COOH reacts with NH_3_ to form –COONH_4_ according to the vibration of –COO^−^ at 1500 cm^−1^, and new peaks at 850 cm^−1^ (marked with #) ascribed to the hydrogen-bonded NH_3_.^19^ From the NH_3_-TPD detection curve in Fig. 3c, the most ammonia was released from PAA–P4VP when the temperature reached 78 °C, while the NH_3_ desorption peak occurred at 112 °C for PAA, indicating that the NH_3_ release from PAA–P4VP was easier. It should be noted the peaks at about 200 °C, which arise from polymer fragments, collapsed according to the TGA results as shown in Fig. S6;† this indicates that the weights of PAA and PAA–P4VP begin to decline from 207 and 198 °C, respectively. The comparison of the IR spectra of the fresh and recovered samples after NH_3_ desorption in Fig. 3b indicates that the PAA–P4VP could be recovered, and it is probably facilitated by the exothermic CHB formation between PAA and P4VP.^29,56^ The –COONH_4_ remains in PAA for the obvious absorption of COO^−^ at 1540 cm^−1^ (marked with pink shadow), which demonstrates the better properties of PAA–P4VP as an NH_3_ adsorbent as compared to PAA. For 8 consecutive cycles of NH_3_ adsorption–desorption experiments (Fig. 3d), the efficient NH_3_ capacity of PAA–P4VP remained steady while there was an obvious decrease for PAA, which might be due to part of the unrecovered carboxylic acid in PAA. The uptake of NH_3_ and other gases by PAA–P4VP in Fig. S7† shows that there was not considerable CO_2_ or N_2_ adsorption, and the selectivity of NH_3_ as compared to CO_2_ and N_2_ was 54.0 and 408, respectively. As can be seen, the acid–base hybrid polymer PAA–P4VP presented enhanced NH_3_ adsorption and desorption as compared with PAA and high selectivity for NH_3_, which indicate the potential application of acid–base polymers as NH_3_ adsorbents.

### The effects of CHBs on the NH_3_ uptake of PAA–P4VP

To investigate the effects of P4VP on the NH_3_ uptake of PAA–P4VP, other complexes including PAA-BPY and PAA-PS were synthesized (Fig. S8a†). PS has the same chainlike structure like P4VP but without the Lewis base moieties, while BPY has the same Lewis base moieties as P4VP. The PAA-BPY and PAA-PS are non-porous as seen from the SEM in Fig. S8b,† which indicates that the Lewis base moieties and the chainlike structure are important for forming porous complexes. The FT-IR spectra in Fig. S9a† show a blue shift of *ν*(COOH) when PAA reacts with BPY but it was less affected by PS, indicating the formation of the CHBs between PAA and BPY. The NH_3_ uptake properties from Fig. S10† show that the adsorption capacity and desorption of PAA-PS are close to those of PAA, indicating that there was no obvious improvement in the NH_3_ uptake of PAA for the mixture of PS. The NH_3_ capacity of PAA-BPY was 13.0 mmol g^−1^ and just 1.5 mmol NH_3_ g^−1^ remained after desorption under vacuum at 80 °C for 90 min. The superior properties of PAA-BPY as compared to PAA-PS indicate that the insertion of Lewis base moieties benefits NH_3_ uptake capacity and desorption, and the competitive formation of CHBs between carboxylic acid and pyridine groups, which implies that the hybrid polymers are excellent ammonia sorbents.

### NH_3_ uptake of acid–base hybrid polymers

**Fig. 4:**
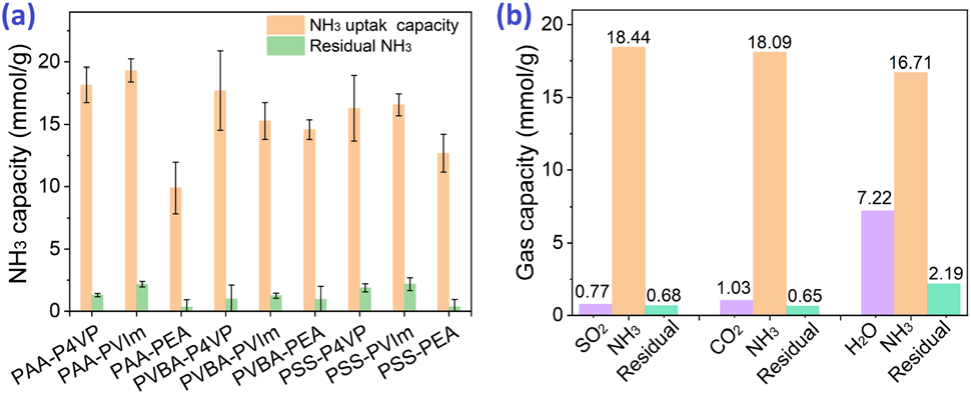
(a) NH_3_ uptake capacity of the acid-basic hybrid polymers at 25 °C and 1 bar. (b) Staged adsorption of 5% SO_2_ and NH_3_, CO_2_ and NH_3_, as well as 3.1% H_2_O and NH_3_ of PAA–P4VP at 25 °C. The residual gases were obtained after desorption in a vacuum at 80 °C for 100 min. The remaining capacity of adsorbate after desorption was converted into NH_3_ gas.

Based on the strategy of developing acid–base hybrid polymers for improved NH_3_ uptake, hybrid acid–base polymers constructed with PAA and PVBA as acid polymers, P4VP and PVIm as Lewis base polymers were synthesized to investigate the effects of the interactions between acid and base moieties on NH_3_ uptake properties. These hybrid polymers were used for NH_3_ uptake at 1 bar and 25 °C; their NH_3_ capacity was 15.8–19.3 mmol g^−1^ from Fig. 4a and most of the fixed NH_3_ could be desorbed under vacuum at 80 °C for 100 min. Simultaneously, the N_2_ and CO_2_ adsorption by the hybrid polymers was also measured at 1 bar and 25 °C, which showed that tiny amounts of CO_2_ but non-considerable N_2_ could be adsorbed, as shown in Table 2. The selectivity of NH_3_ as compared to CO_2_ and N_2_ was 25.4–54.0 and 254–1068, respectively, which suggests the potential for the efficient separation of NH_3_ from these mixture gases. Besides, some interfering gases are inevitably present in industry, and the effects of the adsorbed CO_2_, SO_2_, and H_2_O on NH_3_ uptake were investigated. Fig. 4b shows that 18.44 mmol g^−1^ NH_3_ would be fixed after 0.77 mmol g^−1^ SO_2_ adsorption of PAA–P4VP, and the same phenomenon for CO_2_, which indicates no obvious effect of the acid gas on the NH_3_ uptake by PAA–P4VP. There was a considerable H_2_O uptake capacity of 7.22 mmol g^−1^ by PAA–P4VP but the subsequent ammonia adsorption capacity was reduced to 16.71 mmol g^−1^, likely due to the active hydrogen bonding sites being occupied by H_2_O.

**Fig. 5:**
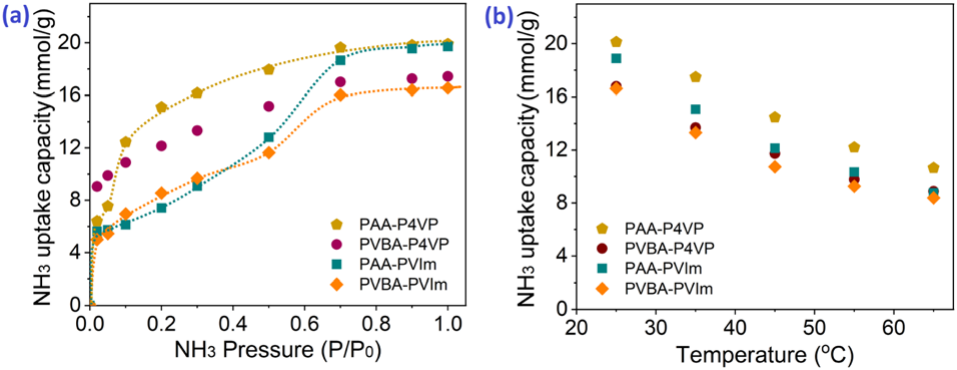
NH_3_ uptake capacity of acid-basic hybrid polymers at various temperatures under 1 bar NH_3_ pressure (a) and under various pressures at 25 °C (b).

The capacity of acid-basic hybrid polymers is susceptible to ammonia pressure and temperature; from Fig. 5, the NH_3_ capacity decreases along with the increase in temperature and decrease in NH_3_ pressure, which indicates that the NH_3_ would be desorbed with the variation of temperature and pressure. It should be noted that there were uptake plateaus at *P*/*P*_0_ = 0.05 of PAA–P4VP and *P*/*P*_0_ = 0.2 of PAA-PVIm and PVBA-PVIm, and their stepwise uptake of approximately 6 mmol g^−1^ from Fig. 5a, corresponding to about 1 equivalent of NH_3_ per mol –COOH for the acid–base reaction to –COONH_4_. Another 10–13 mmol g^−1^ NH_3_ uptake of these hybrid polymers probably contributed to the hydrogen interaction.

### The analysis of NH_3_ uptake of acid–base hybrid polymers

**Fig. 6:**
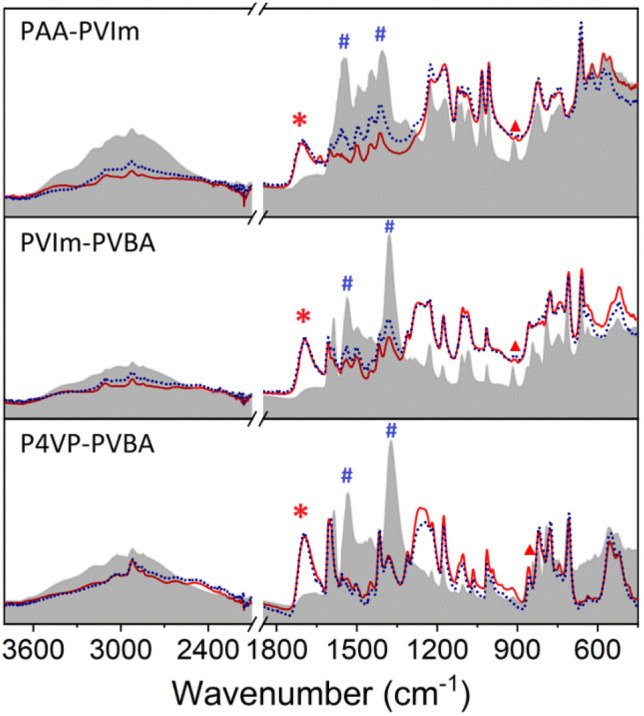
Comparison of the partial IR spectra of hybrid polymers (red solid line) with their NH_3_ saturated (gray background) and desorbed states (blue dotted lines).

The IR spectra of the hybrid polymers compared with their NH_3_ saturated state are shown in Fig. 6. The increase in absorption at about 3400 cm^−1^ was due to the N–H stretching of adsorbed ammonia. Another characteristic peak at about 1470 cm^−1^ increased after ammonia absorption, which can be attributed to the symmetric deformation of the ammonium ion.^57,58^ Meanwhile, the disappearance of *ν*_s_(COOH) at about 1710 cm^−1^ (marked with *) and the increase in *ν*_s_(COO^−^) and *ν*_as_(COO^−^) at about 1520 cm^−1^ and 1350 cm^−1^ (marked with #) indicate the carboxylic acid of hybrid polymers forming the carboxylate salt.^59^ These results support the formation of NH_4_^+^ from the reaction of NH_3_ with the proton of hybrid polymers.^60^ A new peak at about 910 cm^−1^ (marked with ▼) is ascribed to the hydrogen bonding of NH_3_ from PAA-PVIm and PVBA-PVIm, while that at 850 cm^−1^ was due to PVBA–P4VP.^19^ The results claim the hybrid polymers from carboxylic acid polymers and Lewis base polymers achieved high NH_3_ uptake capacity, which is attributed to the cooperative acid–base interaction and hydrogen bonding interactions.

The properties of NH_3_ desorption from the hybrid acid–base polymers were investigated through NH_3_-TPD measurements. The results presented in Fig. S11† indicate NH_3_ desorption peak before 100 °C for all these hybrid polymers, the active energy of desorption of 103.2–107.6 kJ mol^−1^ as listed in Table 1, which belonged to the NH_3_ released from the NH_3_ fixed by acid–base interactions. The ammonia trapped by PVBA–P4VP and PVBA-PVIm would be released completely within 200 °C, indicating the feasible desorption of NH_3_ from these hybrid polymers. The IR spectra of the hybrid polymers after the NH_3_-TPD test compared with the pristine sample in Fig. 6 indicate that carboxylic acid would be recovered after NH_3_ desorption. The reusability of these hybrid polymers for NH_3_ uptake was further investigated. Fig. S12–14† show that the NH_3_ uptake capacity did not decrease within 6 cycles of consecutive NH_3_ uptake and desorption. About 2.8 mmol g^−1^ NH_3_ remained in PAA-PVIm (2.3 mmol g^−1^ NH_3_ remained in PVBA-PVIm) after desorption, which would not affect the subsequent NH_3_ adsorption. The reversibility of these hybrid polymers indicates that the acidic polymer hybrid with appropriate basic polymer is one of the designable strategies to promote NH_3_ desorption and the recovery of hybrid polymers.

## Conclusions

The simple acid–base hybrid polymer PAA–P4VP presents a porous structure and superior NH_3_ uptake properties as compared to PAA for synergetic NH_3_ capture through acid–base and CHB interactions. Inspired by this result, a series of mesoporous acid–base hybrid polymers presenting surface areas of 11.2 to 23.6 m^2^ g^−1^ were synthesized according to the predicted interactions between the acidic and basic moieties. These acid–base hybrid polymers were used for efficient NH_3_ uptake with the capacity of 15.8–19.3 mmol NH_3_ g^−1^ through the cooperative acid–base reaction and hydrogen bonding interactions. The trapped NH_3_ can be released at 80 °C under vacuum along with the reformation of CHBs between the –COOH and Lewis base moieties. This indicates that the incorporation of the basic moiety is a feasible method for improving the NH_3_ uptake and desorption of materials with the carboxylic acid groups.

## Author contributions

Conceptualization, Xiaoyan Luo and Congmin Wang; data curation, Yibang Liu; formal analysis, Xiaoyan Luo and Yibang Liu; funding acquisition, Xiaoyan Luo; investigation, Xiaoyan Luo and Yibang Liu; methodology, Yibang Liu and Mingxing Li; project administration, Congmin Wang; resources, Ling Ye, Xuegong Cao and Congmin Wang; software, Xuegong Cao; supervision, Xiaoyan Luo and Congmin Wang; validation, Yibang Liu, Mingxing Li and Renhui Ling; writing – original draft, Xiaoyan Luo; writing – review & editing, Congmin Wang.

## Conflicts of interest

There are no conflicts to declare.

## Supplementary Material

RA-013-D3RA05346F-s001
