# Supplementary material for: Porous acid–base hybrid polymers for enhanced NH3 uptake with assistance from cooperative hydrogen bonds
Source: RSC Adv. 2023 Oct 2;13(41):28729–35. doi: 10.1039/d3ra05346f (PMC10543883; doi:10.1039/d3ra05346f)
Supplement: RA-013-D3RA05346F-s001 [file RA-013-D3RA05346F-s001.pdf]

Electronic Supplementary Material (ESI) for RSC Advances.
This journal is © The Royal Society of Chemistry 2023

Supporting Information

Porous Acid-Base Hybrid Polymers for Enhanced NH_3 Uptake with the Assist from Cooperative Hydrogen Bonds

Xiaoyan Luo [a,*], Yibang Liu [a], Mingxing Li[a], Renhui Ling [a], Ling Ye [a], Xuegong Cao [a], Congmin Wang [b,*].

a. Xiamen Key Laboratory of Optoelectronic Materials and Advanced Manufacturing, Key Laboratory of Molecular Designing and Green Conversions (Fujian Province University), College of Materials Science and Engineering, Huaqiao University, Xiamen 361021 (P.R. China).

b. Department of Chemistry, Center of Chemistry for Frontier Technologies, Zhejiang University, Hangzhou 310027 (P. R. China).

* chemistrylxy@163.com; chewcm@zju.edu.cn.

This file contains the experiment section, 12 figures, and 2 tables.

1. Experimental section

1.1. Materials

NH_3 (99.999 %) was supplied by Foushan Kodi Gas Chemical Industry Co., Ltd. 4,4'-Bipyridine (BPY, 98 %), 4-vinylpyridine (4VP, 96 %, containing 80-120 ppm hydroquinone stabilizer), poly(acrylic acid) (PAA, 99%, Mw 450000 g/mol), polystyrene (food grade), polystyrene (PS, 30wt%, Mw~350000 g/mol), 4-Vinylbenzoic acid (VBA, 95%, 100ppm BHT) were purchased by Shanghai Aladdin biochemical technology Co., Ltd. 2,2'-azobis(2-methylpropionitrile) (AIBN) was provided by Shanghai Macklin Biochemical Co., Ltd. Hydrochloric acid aqueous solution (36-38 wt%), sodium hydroxide (96%) were purchased from Sinopharm Chemical Reagent Co., Ltd. 4-vinylpyridine was re-steamed before used. Other reagents were used without further treatment, unless otherwise noted.

1.2. Synthesis of hybrid polymers

The synthesis and characterization of P4VP and PVIm were described in our previous work in detail.[1] The molecular weight of P4VP and PVIm are about 116900 and 49400 g/mol, respectively, according to the viscosity method.

1.2.1. Synthesis of PVBA. 4-Vinylbenzoic acid (VBA, 10.0 g) and 50 mL deionized water were put in a 150 mL three-seater flask, 0.16g AIBN was added in the solution after bubbling through N_2 flow for 0.5 hour to drive the oxygen in the solution away. The solution was stirred under protection of N_2 for 12 hours at 60 ^oC, the obtained white solid powder was filtrated and dried in 80 ^oC vacuum. Yeild, 86.3%. The IR and ^1H NMR spectra of the synthesized PVBA were exhibited in Figure S1a and Figure S1b with the comparison of VBA. IR (cm^{-1}): 3500-3100 (υ_{OH}), 3022 (υ_{CH} of benzene ring), 2917, 2663, and 2536 (υ_{CH} of methylene and methyne), 1687 and 1279 (υ_{COOH}), 1607 and 1575 ($\upsilon_{benzene\ ring}$). ^1H NMR (d_6-DMSO, ppm): 1.24 (1H, CH), 1.44 (2H, CH_2), 6.63 (2H, CH in benzene ring), 7.65 (2H, CH in benzene ring), 12.79 (1H, COOH). he molecular weight of PVBA is about 306000 g/mol according to the viscosity method.

1.2.2. Synthesis of PAA-P4VP. 1.44 g PAA (containing 20 mmol carboxylate acid) was dissolved in 40 mL mixed solvent while 2.10 g P4VP (containing 20 mmol pyridine) was dissolved in another 40 mL mixed solvent, where the mixed solvent is methanol and deionized water (V:V=1:1). Add the PAA solution into P-4VP solution drop by drop and then stirring for 3 h at room temperature, there was white floccules precipitated out. The white floccules were obtained through centrifugation and dealt with freeze drying, and then placed into the vacuum drying oven at 80°C for 24 h. Yield, 87.8%.

Synthesis of PAA-PVIm, PVBA-P4VP, PVBA-PVIm, and PAA-BPY were like PAA-P4VP, their yield was 92.5%, 51.4%, 60.8%, 81.3%, 76.4%, and 84.7%, respectively.

1.2.3. Synthesis of PAA-PS.1.44 g PAA was dissolved in 40 mL H_2O while 2.08 g PS (containing 20 mmol benzene ring) was dissolved in 40 mL tetrahydrofuran. Add the PAA aqueous solution into PS solution drop by drop and then stirring for 3 h at room temperature. Most of the solvent in the solution was removed by rotary evaporation, and the obtained white solid was dried at 80 °C in a vacuum oven for 24 h. Yield, 96.4%.

1.3. Characterization of acid frustrated hybrid polymers.

^1H NMR spectra was measured using a Bruker spectrometer (500 MHz) with tetramethyl silane (TMS, 0.00 ppm) as an internal standard. Fourier transform infrared spectroscope (Nicolet IS 50 FT-IR) was recorded with the attenuated total reflectance (ATR) module in the range of 400-4000 cm^{-1}. 2D correlation FT-IR spectra were got by 2D software based on FT-IR spectra and the analysis was according to the so-called Noda's rule.[2] Scanning Electron Microscopy (SEM, JSM-7610F Plus) were used for morphological characterization of the gold-sprayed sample. Thermogravimetric analysis (TGA) was performed with a ramp rate of 10 °C/min from 60 °C to 600°Cunder Ar atmosphere by using Shimadzu DTG-60H. Elemental analyses for C, H, and N were performed at the Vario EL Cube Element analyzer (EA). The NH_3-TPD of hybrid polymers was detected by PCA-1200 chemical adsorption instrument with the nitrogen flow of 30 mL/min at the programmed heating rate of 2 °C/min, where the samples were NH_3 saturated. N_2 adsorption isotherm measurements were performed on a micromeritics 3FLEX surface characterization measurement at 77 K.

1.4. NH_3 absorption and desorption experiment

NH_3 and CO_2 uptake performance of the hybrid polymers were measured by weighing method through open method as presented in Figure S14. The open method was operated with controlled ammonia or CO_2 gas flow of 40 sccm through the polymers until the weight of the sample keep steady within 5 min. The effect of NH_3 partial pressure for open uptake was investigated by altering the composition of NH_3 and N_2. The NH_3 desorption from the hybrid polymers were carried out under vacuum at 80 °C and 0.1 kPa until the weight maintained constant. The coefficient of selectivity for NH_3/CO_2 and NH_3/N_2 were calculated according to the equation $S_{1/2} = \frac{C_1}{C_2}$, where 1 and 2 are two gas, $S_{1/2}$ is the adsorption selectivity of gas 1 over

gas 2, c_1 and c_2 is the gas adsorption capacity of 1 and 2 (mmol/g) at 1bar and 25 °C. Thereinto, the CO_2 and N_2 adsorption were measured on a micromeritics 3FLEX surface characterization measurement at 25 °C.

The effect of the containing water on NH_3 uptake of hybrid polymers was tested in the apparatus as shown in our previous work.[3] 30 sccm N_2 flow passed through 25°C water (saturated vapor pressure is 3.169 kPa) and hybrid polymer in turn, where the mixture gas passed through the hybrid polymer was moist N_2 steam containing 3.1% H_2O. The weight increase of hybrid polymer was considered as H_2O adsorption. The SO_2 uptake of hybrid polymer was tested using the apparatus shown in Figure S15, 30 sccm mixture flow with 5% SO_2 and 95% N_2 passed through hybrid polymer at 25°C, the weight increase of hybrid polymer was considered as SO_2 adsorption.

2. Supporting Figures.

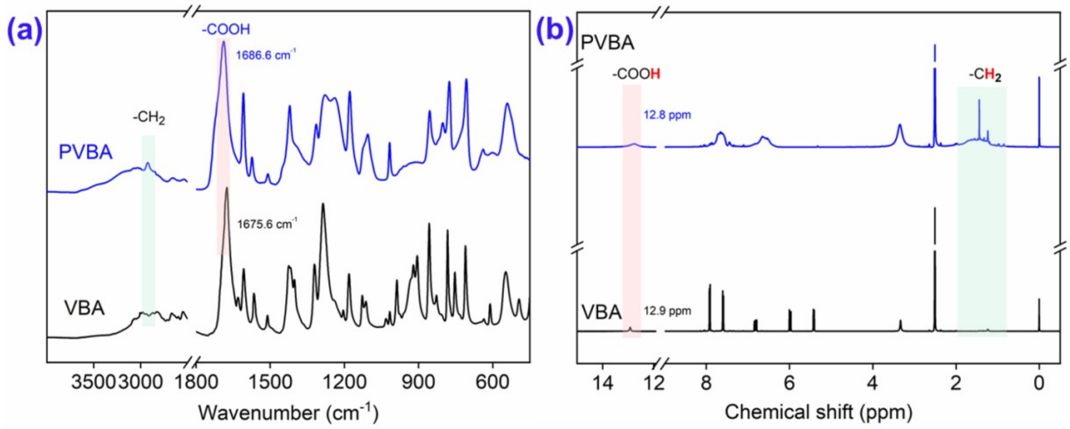

Figure S1. The comparison of IR and ^{1}H NMR spectra of VBA and PVBA.

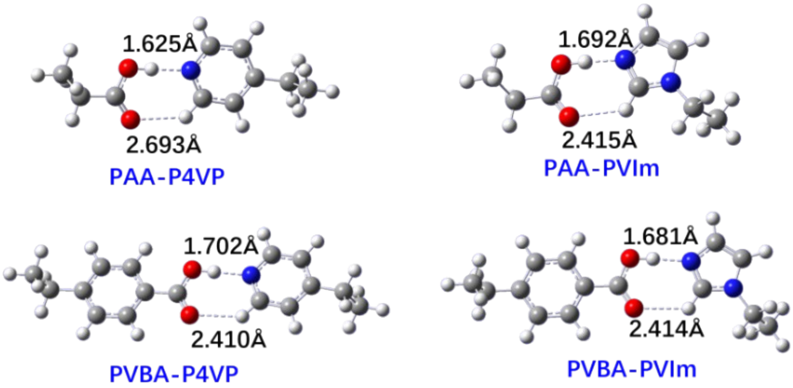

Figure S2. Optimized structure of the unit of hybrid polymers.

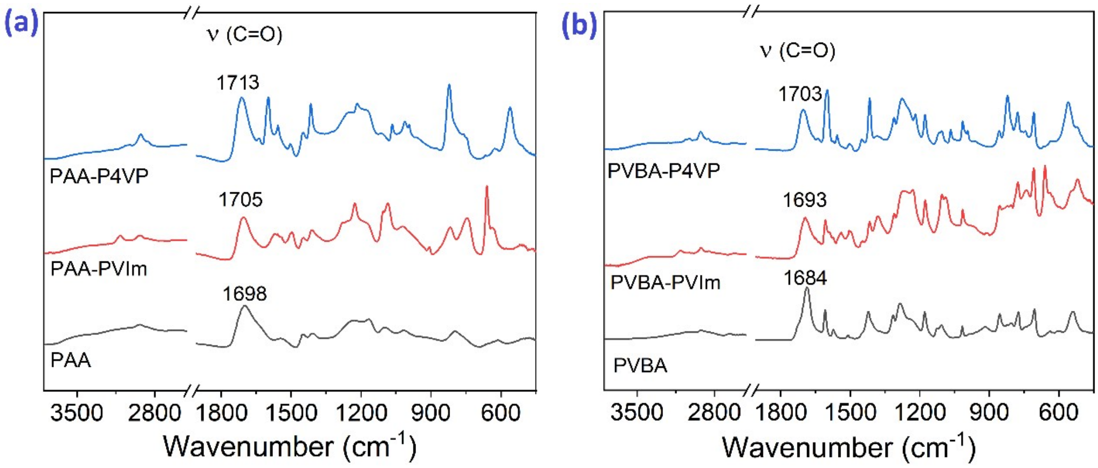

Figure S3. Comparison of the IR spectra of hybrid polymers with PAA and PVBA.

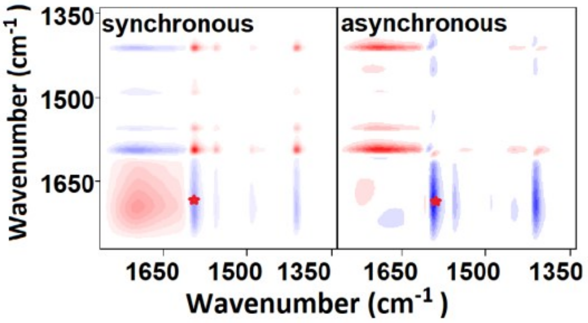

Figure S4. 2D corrected IR spectra of PAA-P4VP with isochronous content of PAA.

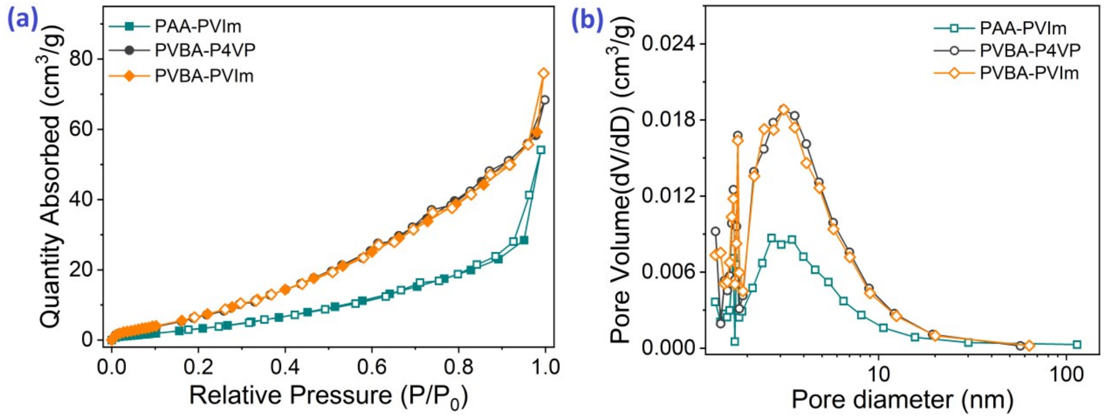

Figure S5. (a) N_2 adsorption isotherm hybrid polymers at 77K. (b) The pore distribution of hybrid polymers.

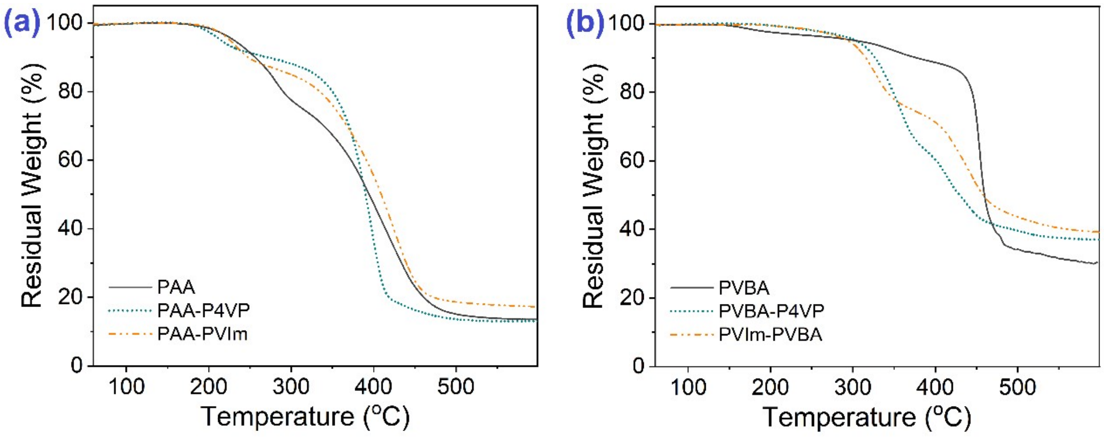

Figure S6 TGA curves of hybrid polymers from 60 to 600 ^{o}C with temperature increase ratio of 10 ^{o}C/min under Ar flow.

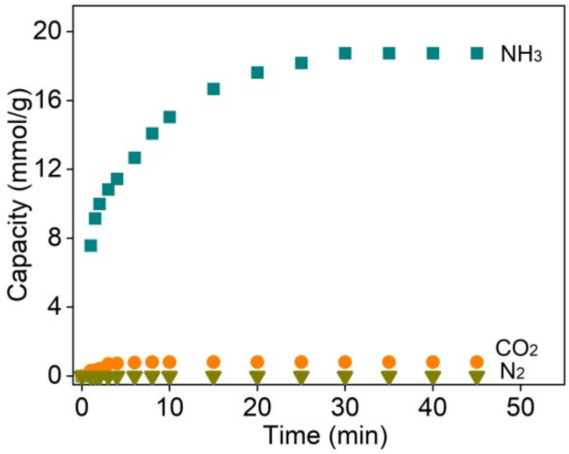

Figure S7. NH_3, CO_2, and N_2 uptake of PAA-P4VP at 298K and under 1 bar gas pressure.

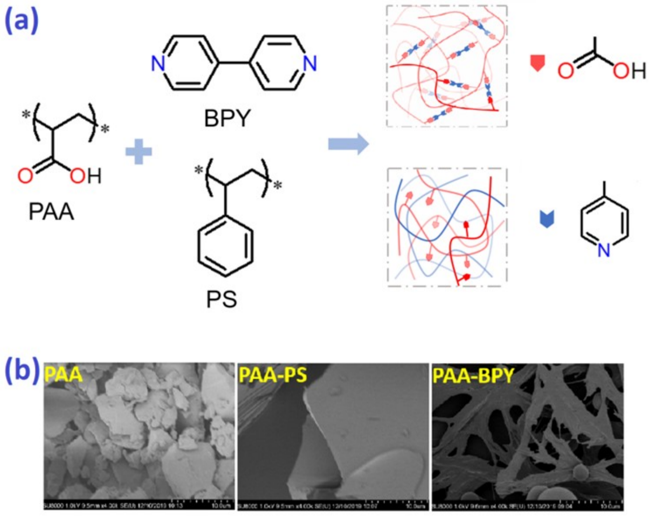

Figure S8. (a) Structure of the agents used for synthesizing hybrid polymers. (b) SEM images of PAA, PAA-PS, and PAA-BPY.

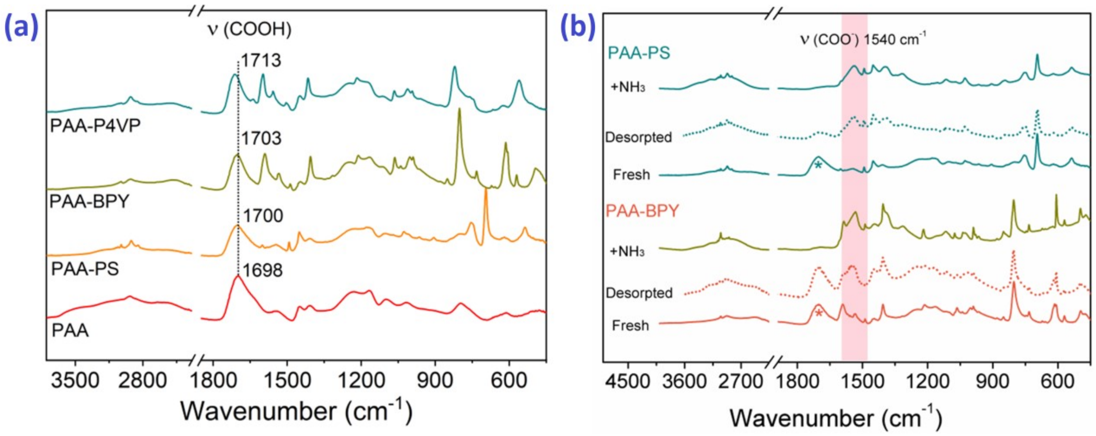

Figure S9. Comparison of partial IR spectra of PAA-BPY, and PAA-PS with their NH_3 saturated and desorbed states.

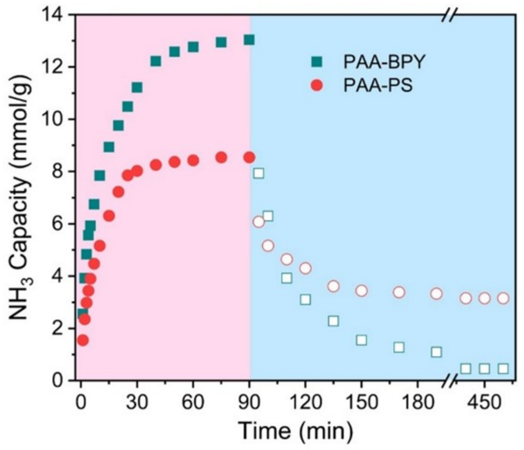

Figure S10. (a) The NH_3 uptake and desorption of PAA-PS and PAA-BPY. NH_3 uptake and release at 25 °C, 1bar and 80 °C vacuum, respectively.

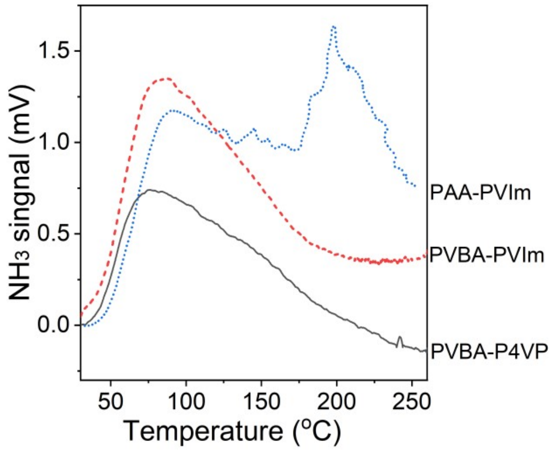

Figure S11. NH_3-TPD curve of hybrid polymers PAA-PVIm, PVBA-PVIm, and PVBA-P4VP.

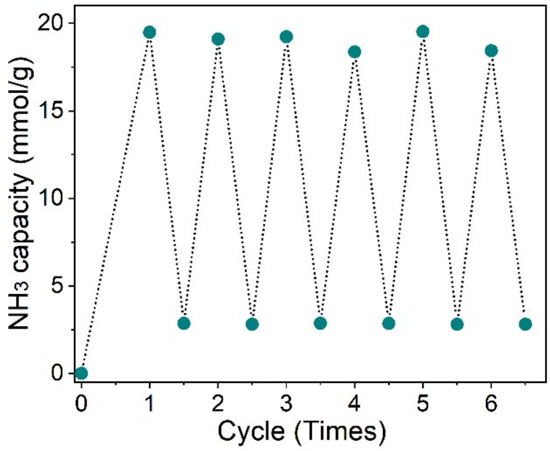

Figure S12. 6 consecutive NH_3 uptake capacity of PAA-PVIm at 25 °C and desorption under 80 °C vacuum for 90 min.

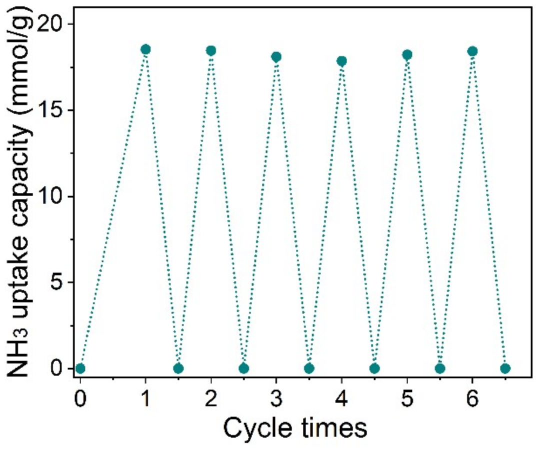

Figure S13. 6 consecutive NH_3 uptake capacity of PVBA-P4VP at 25 ^oC and desorption under 80 ^oC vacuum for 90 min.

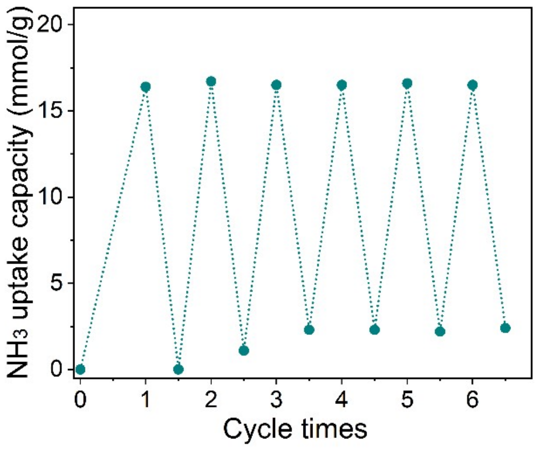

Figure S14. 6 consecutive NH_3 uptake capacity of PVBA-PVIm at 25 ^oC and desorption under 80 ^oC vacuum for 90 min.

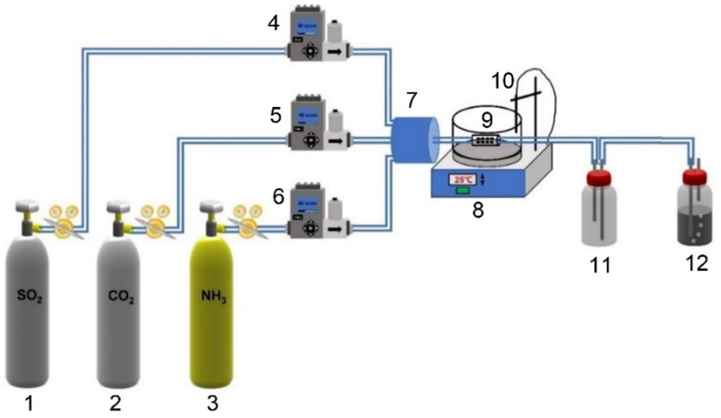

Figure S15. Ammonia adsorption device. 1, SO_2 cylinder. 2, CO_2 cylinder. 3, NH_3 cylinder. 4, 5, and 6, gas velocity controlier.7. gas mixer. 8. temperature controlling system.9 sample cell. 10. temperature detector. 11, safely fack.12. exhaust gas absorption bottle containing dilute sulfuric acid solution.

3. Supporting Tables.

Table S1. The element content of acid-basic hybrid complexes.

Sample	Element Content (%)		
	C	N	H
PAA-P4VP	62.60 (67.80)	7.09 (7.91)	6.52 (6.21)
PAA-PVIm	52.18 (57.83)	15.00 (16.87)	6.68 (6.02)
PVBA-P4VP	72.81 (74.60)	5.61 (5.81)	5.57 (6.22)
PVBA-PVIm	65.31 (69.42)	10.00 (11.57)	5.59 (5.78)

The data in the bracket is the calculated element content corresponding to the molar ratio of acid and base moieties as 1:1.

Table S2. Comparison of NH_3 uptake properties of various sorbents with acidic sites.

Capture Agent		NH_3 Capacity (mmol/g)	Adsorption condition	Desorption condition	Refs
Hybrid polymers	PAA-P4VP	18.2	298K, 1bar	353 K and vacuum	This Work
Hybrid polymers	PAA-PVIm	19.3	298K, 1bar	353 K and vacuum	This Work
COFs	P1-PO_3H_2	18.7	298K, 1bar	――――――	Jeffrey R. Long[4]
COFs	BPP-5	17.7	298K, 1bar	298K and vacuum	Jeffrey R. Long[5]
COFs	TpBD-$(SO_3H)_2$	11.5	298K, 1bar	――――――	Baiyan Li[6]
COFs	$[COOH]_{33}$-COF	9.1	298K, 1bar	473K and vacuum	Ye Yuan[7]
POPs	1TCS	8.5	298K, 1bar	418K and 1bar	Chang S. Hong[8]
polymer	PIM-1-COOH	12.2	298K, 1bar	298K and vacuum	Omar K. Farha[9]
polymer	SOMPs	15.1	298K, 1bar	425K and vacuum	Lilong Jiang[10]
polymer	PAA	10.7	298K, 1bar	353K and vacuum	Jeffrey R. Long[11]
polymer	PVBA-2.0AA	8.8	298K, 1bar	353K and vacuum	Kuan Huang[12]
polymer	PIM-1-COOH	12.2	298K, 1bar	RT and vacuum	Omar K. Farha[13]
MOFs	UiO-66-IL	13.3	298K, 1bar	393K and 1bar	Yunlei Shi[14]
MOFs	NU-300	8.3	298K, 1bar	368K and vacuum	Omar K. Farha[15]
MOFs	MFM-303(Al)	9.9	273K, 1bar	353K and vacuum	Martin Schröder[16]
MOFs	Ni_acryl_TMA	23.54	298K, 1bar	high vacuum	Chang S. Hong[17]
MOFs	UiO-ox	2.5	293K, 2000 ppm	-	Joseph T. Hupp[18]
MOFs	UiO-66-$(COOH)_2$	2.83	293K, 2000 ppm	-	Krista S. Walton[19]
DESs	1GA-3XYL	13.2	303K, 1bar	333K and vacuum	Baoyou Liu[20]
DESs	2MAA-1tetrazole	8.0	313K, 1bar	353K and vacuum	Kuan Huang[21]
DESs	1[ImH]Cl-2Gly	12.9	298K, 1bar	333K and vacuum	Kuan Huang[22]
DESs	1ChCl-5PhOH-4EG	9.6	298K, 1bar	333K and vacuum	Hailong Peng[23]
DESs	1EaCl-2Gly	9.6	298K, 1bar	353K and vacuum	Yong Liu[24]
DESs	1[Im][SCN]–3EG	13.7	303K, 1bar	343K, N_2 sweeping	Dongshun Deng[25]

Continued Table S2. Comparison of NH_3 uptake properties of various sorbents with

acidic sites.

DESs	$2NH_4SCN$-3G	13.1	303K, 1bar	333K, N_2 sweeping	Dongshun Deng[26]
DESs	$0.1MgCl_2$-1Res-2EG	12.1	313K, 1bar	353K and vacuum	Jingai Hao[27]
DESs	1Atri-5Res-4Gly	11.4	313K, 1bar	353K and vacuum	Jingai Hao[28]
DESs	1EaCl-4Res-5Gly	10.6	313K, 1bar	353K, N_2 sweeping	Jingai Hao[29]
DESs	1Im-1Res	9.1	313K, 1bar	353K and vacuum	Jingai Hao[30]
DESs	1Tri-3Gly	6.8	313K, 1bar	343K, N_2 sweeping	Dongshun Deng[31]
DESs	$[C_1NH_3][Zn_3Cl_7]$@FDU	25.2	298K, 1bar	423K	Zhiyong Li[32]
DESs	1EaCl-1Res	9.0	298K, 1bar	353K and vacuum	Hailong Peng[33]
ILs	$[N_{111,1COOH}][Tf_2N]$	~6.1	298K, 1bar	————	Takashi Makino[34]
ILs	[EtA][SCN]	17.4	303K, 1bar	343K, N_2 sweeping	Dongshun Deng[35]
ILs	$[Bmim]_2$ $[Co(NCS)_4]$	10.6	303K, 1bar	353K, N_2 sweeping	Xiangping Zhang[36]
ILs	$[2PyH][Tf_2N]$	10.1	303K, 1bar	323K and vacuum	Jinqing Lin[37]
ILs	$[Bmim]_2[CuCl_4]$	10.1	303K, 1bar	353K, N_2 sweeping	Xiangping Zhang[38]
ILs	$[2\text{-}Mim][NTf_2]$/AC-980	4.0	303K, 1bar	353K, N_2 sweeping	Xiangping Zhang[39]
ILs	$[2\text{-}Mim][Li(NTf_2)_2]$	10.8	313K, 1bar	353K and vacuum	Shaojuan Zeng[40]
ILs	$[4\text{-}MeOHPy][NTf_2]$	8.8	313K, 1bar	353K, N_2 sweeping	HongShuai Gao[41]
ILs	$[2\text{-}mPy][NTf_2]$	8.2	313K, 1bar		Shaojuan Zeng[42]
Biochar	$CuSiO_3$/PBC	6.4	298K, 1bar	323K and vacuum	Shaojun Yuan[43]
Coal	CT-5-300	3.1	298K, 1bar	————	Deli Chen[44]
Coal	BC-300	2.9	298K, 1bar	————	Deli Chen[45]
Carbon	MA2ox	1.0	298K, 1bar	————	Silvestre-Albero[46]

4. Reference.

(1) Luo, L.; Liu, Y.; Wu, Z.; Liu, J.; Cao, X.; Lin, J.; Ling, R.; Luo, X.; Wang, C. Macromolecular-metal complexes induced by Co(II) with polymer and flexible ligands for ammonia uptake compared with MOFs. *Chem Eng J* **2022**, *448*, 137626.

(2) I. Noda, Y. O. Two-Dimensional Correlation Spectroscopy. Application in Vibrational and Optical Spectroscopy. *Chichester, UK* **2004**. John Wiley & Sons, Ltd.

(3) Luo, L.; Li, J.; Chen, X.; Cao, X.; Liu, Y.; Wu, Z.; Luo, X.; Wang, C. Superhigh and reversible NH_3 uptake of cobaltous thiocyanate functionalized porous poly ionic liquids through competitive and cooperative interactions. *Chem Eng J* **2022**, *427*, 131638.

(4) Barin, G.; Peterson, G. W.; Crocella, V.; Xu, J.; Colwell, K. A.; Nandy, A.; Reimer, J. A.; Bordiga, S.; Long, J. R. Highly effective ammonia removal in a series of Bronsted acidic porous polymers: investigation of chemical and structural variations. *Chem Sci* **2017**, *8* (6), 4399-4409.

(5) Van Humbeck, J. F.; McDonald, T. M.; Jing, X.; Wiers, B. M.; Zhu, G.; Long, J. R. Ammonia capture in porous organic polymers densely functionalized with Bronsted acid groups. *J Am Chem Soc* **2014**, *136* (6), 2432-2440.

(6) Li, J.; Xiao, Y.; Shui, F.; Yi, M.; Zhang, Z.; Liu, X.; Zhang, L.; You, Z.; Yang, R.; Yang, S.; et al. Extremely Stable Sulfuric Acid Covalent Organic Framework for Highly Effective Ammonia Capture. *Chinese J Chem* **2022**, *40* (20), 2445-2450.

(7) Yang, Y.; Faheem, M.; Wang, L.; Meng, Q.; Sha, H.; Yang, N.; Yuan, Y.; Zhu, G. Surface Pore Engineering of Covalent Organic Frameworks for Ammonia Capture through Synergistic Multivariate and Open Metal Site Approaches. *ACS Cent Sci* **2018**, *4* (6), 748-754.

(8) Kang, D. W.; Kang, M.; Moon, M.; Kim, H.; Eom, S.; Choe, J. H.; Lee, W. R.; Hong, C. S. PDMS-coated hypercrosslinked porous organic polymers modified via double postsynthetic acidifications for ammonia capture. *Chem Sci* **2018**, *9* (33), 6871-6877.

(9) Jung, D.; Chen, Z.; Alayoglu, S.; Mian, M. R.; Goetjen, T. A.; Idrees, K. B.; Kirlikovali, K. O.; Islamoglu, T.; Farha, O. K. Postsynthetically Modified Polymers of Intrinsic Microporosity (PIMs) for Capturing Toxic Gases. *ACS Appl Mater Interf* **2021**, *13* (8), 10409-10415.
(10) Kan, X.; Liu, Z.; Liu, F.; Li, F.; Chen, W.; Yi, X.; Zheng, A.; Jiang, L.; Xiao, F.-S. Sulfonated and ordered mesoporous polymers for reversible adsorption of ammonia: Elucidation of sequential pore-space diffusion. *Chem Eng J* **2023**, *451*, 139085.
(11) Lee, J. W.; Barin, G.; Peterson, G. W.; Xu, J.; Colwell, K. A.; Long, J. R. A Microporous Amic Acid Polymer for Enhanced Ammonia Capture. *ACS Appl Mater Interf* **2017**, *9* (39), 33504-33510.
(12) Zhang, J.; Ma, Y.; Wu, W.; Cai, Z.; Cao, Y.; Huang, K.; Jiang, L. Carboxylic functionalized mesoporous polymers for fast, highly efficient, selective and reversible adsorption of ammonia. *Chem Eng J* **2022**, *448*, 137640.
(13) Jung, D.; Chen, Z.; Alayoglu, S.; Mian, M. R.; Goetjen, T. A.; Idrees, K. B.; Kirlikovali, K. O.; Islamoglu, T.; Farha, O. K. Postsynthetically Modified Polymers of Intrinsic Microporosity (PIMs) for Capturing Toxic Gases. *Acs Appl Mater Inter* **2021**, *13* (8), 10409-10415.
(14) Wang, Y.; Shi, Y.; Li, Z.; Wang, H.; Qiu, J.; Xuan, X.; Wang, J. Defects in a Metal–Organic Framework Fabricated by Carboxy-Functionalized Ionic Liquids for Enhancing NH_3 Uptake. *Acs Sustain Chem Eng* **2022**, *10* (37), 12457-12465.
(15) Chen, Y.; Zhang, X.; Ma, K.; Chen, Z.; Wang, X.; Knapp, J.; Alayoglu, S.; Wang, F.; Xia, Q.; Li, Z.; et al. Zirconium-Based Metal–Organic Framework with 9-Connected Nodes for Ammonia Capture. *ACS Appl Nano Mater* **2019**, *2* (10), 6098-6102.
(16) Marsh, C.; Han, X.; Li, J.; Lu, Z.; Argent, S. P.; da Silva, I.; Cheng, Y.; Daemen, L. L.; Ramirez-Cuesta, A. J.; Thompson, S. P.; et al. Exceptional Packing Density of Ammonia in a Dual-Functionalized Metal–Organic Framework. *J Am Chem Soc* **2021**, *143* (17), 6586-6592.
(17) Kim, D. W.; Kang, D. W.; Kang, M.; Choi, D. S.; Yun, H.; Kim, S. Y.; Lee, S. M.; Lee, J.-H.; Hong, C. S. High Gravimetric and Volumetric Ammonia Capacities in Robust Metal–Organic Frameworks Prepared via Double Postsynthetic Modification. *J Am Chem Soc* **2022**, *144* (22), 9672-9683.
(18) DeCoste, J. B.; Demasky, T. J.; Katz, M. J.; Farha, O. K.; Hupp, J. T. A UiO-66 analogue with uncoordinated carboxylic acids for the broad-spectrum removal of toxic chemicals. *New J Chem* **2015**, *39* (4), 2396-2399.
(19) Jasuja, H.; Peterson, G. W.; Decoste, J. B.; Browe, M. A.; Walton, K. S. Evaluation of MOFs for air purification and air quality control applications: Ammonia removal from air. *Chem Eng Sci* **2015**, *124*, 118-124.
(20) Liu, B.; Tian, J. Investigation of Glycolic Acid Natural Deep Eutectic Solvents with Strong Proton Donors for Ammonia Capture and Separation. *Ind Eng Chem Res* **2021**, *60* (30), 11600-11610.
(21) Li, Z.-L.; Zhong, F.-Y.; Zhou, L.-S.; Tian, Z.-Q.; Huang, K. Deep Eutectic Solvents Formed by N-Methylacetamide and Heterocyclic Weak Acids for Highly Efficient and Reversible Chemical Absorption of Ammonia. *Ind Eng Chem Res* **2020**, *59* (5), 2060-2067.
(22) Ma, Y.; Zhang, J.; Huang, K.; Jiang, L. Highly efficient and selective separation of ammonia by deep eutectic solvents through cooperative acid-base and strong hydrogen-bond interaction. *J Mol Liq* **2021**, *337*, 116463.
(23) Zhong, F.-Y.; Peng, H.-L.; Tao, D.-J.; Wu, P.-K.; Fan, J.-P.; Huang, K. Phenol-Based Ternary Deep Eutectic Solvents for Highly Efficient and Reversible Absorption of NH_3. *ACS Sustain Chem Eng* **2019**, *7* (3), 3258-3266.
(24) Jiang, W.-J.; Zhong, F.-Y.; Liu, Y.; Huang, K. Effective and Reversible Capture of NH_3 by Ethylamine Hydrochloride Plus Glycerol Deep Eutectic Solvents. *ACS Sustain Chem Eng* **2019**, *7* (12), 10552-10560.
(25) Zhou, Z.; Li, K.; Zong, K.; Deng, X.; Deng, D. Investigation of NH_3 absorption by protic imidazolium thiocyanate-based deep eutectic solvents with multiple binding sites and low viscosity. *New J Chem* **2021**, *45* (45), 21108-21115.
(26) Deng, D.; Gao, B.; Zhang, C.; Duan, X.; Cui, Y.; Ning, J. Investigation of protic NH_4SCN-based deep

eutectic solvents as highly efficient and reversible NH3 absorbents. *Chem Eng J* **2019**, *358*, 936-943.
(27) Sun, X.; Wang, Q.; Wu, S.; Zhao, X.; Wei, L.; Li, K.; Hao, J.; Wei, L.; Zhai, S.; An, Q. Metal chlorides-promoted ammonia absorption of deep eutectic solvent. *Int J Hydrogen Energ* **2022**, *47* (36), 16121-16131.
(28) Wang, Q.; Wang, Y.; Sun, X.; Wei, L.; Wei, L.; Zhai, S.; Xiao, Z.; An, Q.; Hao, J. Constructing ternary deep eutectic solvents with multiple sites for ammonia storage. *Int J Hydrogen Energ* **2022**, *47* (80), 34102-34111.
(29) Luo, Q.; Hao, J.; Wei, L.; Zhai, S.; Xiao, Z.; An, Q. Protic ethanolamine hydrochloride-based deep eutectic solvents for highly efficient and reversible absorption of NH_3. *Sep Purif Technol* **2021**, *260*.118240.
(30) Luo, Q.; Wang, Q.; Sun, X.; Wu, H.; Hao, J.; Wei, L.; Zhai, S.; Xiao, Z.; An, Q. Dual-active-sites deep eutectic solvents based on imidazole and resorcinol for efficient capture of NH_3. *Chem Eng J* **2021**, *416*. 129114.
(31) Deng, D.; Duan, X.; Gao, B.; Zhang, C.; Deng, X.; Gong, L. Efficient and reversible absorption of NH_3 by functional azole–glycerol deep eutectic solvents. *New J Chem* **2019**, *43* (29), 11636-11642.
(32) Cao, D.; Li, Z.; Wang, Z.; Wang, H.; Gao, S.; Wang, Y.; Shi, Y.; Qiu, J.; Zhao, Y.; Wang, J. Highly Dispersed Ionic Liquids in Mesoporous Molecular Sieves Enable a Record NH_3 Absorption. *ACS Sustain Chem Eng* **2021**, *9* (48), 16363-16372.
(33) Cheng, N.-N.; Li, Z.-L.; Lan, H.-C.; Xu, W.-L.; Jiang, W.-J.; Huang, K.; Peng, H.-L. Deep eutectic solvents with multiple weak acid sites for highly efficient, reversible and selective absorption of ammonia. *Sep Purif Technol* **2021**, *269*, 118791.
(34) Makino, T.; Kanakubo, M. NH_3 absorption in Brønsted acidic imidazolium- and ammonium-based ionic liquids. *New J Chem* **2020**, *44* (47), 20665-20675.
(35) Deng, D.; Deng, X.; Li, K.; Fang, H. Protic ionic liquid ethanolamine thiocyanate with multiple sites for highly efficient NH_3 uptake and NH_3/CO_2 separation. *Sep Purif Technol* **2021**, *276*, 119298.
(36) Zeng, S.; Liu, L.; Shang, D.; Feng, J.; Dong, H.; Xu, Q.; Zhang, X.; Zhang, S. Efficient and reversible absorption of ammonia by cobalt ionic liquids through Lewis acid–base and cooperative hydrogen bond interactions. *Green Chem* **2018**, *20* (9), 2075-2083.
(37) Luo, X.; Qiu, R.; Chen, X.; Pei, B.; Lin, J.; Wang, C. Reversible Construction of Ionic Networks Through Cooperative Hydrogen Bonds for Efficient Ammonia Absorption. *ACS Sustain Chem Eng* **2019**, *7* (11), 9888-9895.

(38) Wang, J.; Zeng, S.; Huo, F.; Shang, D.; He, H.; Bai, L.; Zhang, X.; Li, J. Metal chloride anion-based ionic liquids for efficient separation of NH_3. *J Clean Prod* **2019**, *206*, 661-669.
(39) Yu, M.; Zeng, S.; Wang, Z.; Hu, Z.; Dong, H.; Nie, Y.; Ren, B.; Zhang, X. Protic Ionic-Liquid-Supported Activated Carbon with Hierarchical Pores for Efficient NH_3 Adsorption. *ACS Sustain Chem Eng* **2019**, *7* (13), 11769-11777.
(40) Shang, D.; Zeng, S.; Zhang, X.; Zhang, X.; Bai, L.; Dong, H. Highly efficient and reversible absorption of NH_3 by dual functionalised ionic liquids with protic and Lewis acidic sites. *J Mol Liq* **2020**, *312*, 113411.
(41) Yuan, L.; Gao, H.; Jiang, H.; Zeng, S.; Li, T.; Ren, B.; Zhang, X. Experimental and thermodynamic analysis of NH_3 absorption in dual-functionalized pyridinium-based ionic liquids. *J Mol Liq* **2021**, *323*, 114601.
(42) Zhang, L.; Liu, C.; Li, Q. B. Molecular simulations of competitive adsorption behavior between CH_4-C_2H_6 in K-illite clay at supercritical conditions. *Fuel* **2020**, *260*, 116358.
(43) Zhang, Y.; Xiao, J.; Zhang, T. C.; Ouyang, L.; Yuan, S. Synthesis of $CuSiO_3$-loaded P-doped porous biochar derived from phytic acid-activated lemon peel for enhanced adsorption of NH_3. *Sep Purif Technol* **2022**, *283*. 120179.
(44) Zhang, W.; Han, B.; Wille, U.; Butterly, C.; He, J.-Z.; Chen, D. Surface modification of coal tailings by thermal air oxidation for ammonia capture. *J Clean Prod* **2022**, *362*, 132525.
(45) Zhang, W.; Han, B.; He, J.-Z.; Chen, D. Modification of bituminous coal by air oxidation to increase

ammonia capture. *J Anal Appl Pyrol* **2020**, *151*. 104930.
(46) Goncalves, M.; Sanchez-Garcia, L.; Oliveira Jardim, E.; Silvestre-Albero, J.; Rodriguez-Reinoso, F. Ammonia removal using activated carbons: effect of the surface chemistry in dry and moist conditions. *Environ Sci Technol* **2011**, *45* (24), 10605-10610.
